# Effect of High-Intensity Gait Training in the Acute Care Setting on Length of Stay and Functional Status in Patients with Stroke

**DOI:** 10.3390/brainsci16101082

**Published:** 2026-10-09

**Authors:** Michael J. Shoemaker, Kaitlyn R. Batchelor, Jozlyn A. Gilman, Rachel S. Miller, Tarah F. Nicholson

**Affiliations:** 1 Department of Physical Therapy and Athletic Training, Grand Valley State University, Grand Rapids, MI 49503, USA; batchelk@mail.gvsu.edu (K.R.B.); gilmanjo@mail.gvsu.edu (J.A.G.); mcdonrac@mail.gvsu.edu (R.S.M.); 2 Department of Rehabilitation Services, Corewell Health West Butterworth Campus, Grand Rapids, MI 49503, USA; tarah.nicholson@corewellhealth.org

**Keywords:** acute stroke, gait training, high intensity, acute care, rehabilitation

## Abstract

**Highlights:**

**What are the main findings?**

- Results from this hypothesis-generating preliminary analysis indicate that receiving high-intensity gait training in the acute care setting was associated with a shorter length of stay compared to a propensity score-matched historical usual care comparator cohort.
- Given there was no difference in change in functional status between groups, the mechanism by which length of stay may be shorter is unclear.

**What is the implication of the main finding?**

- Further study with contemporaneous controlled studies is needed to elucidate the short-term effects of high-intensity gait training in the acute care setting on acute care length of stay.

**Abstract:**

**Background/Objectives**: The feasibility of high-intensity gait training (HIGT) in the acute care setting has only been demonstrated in two studies and its impact on functional status and length of stay (LOS) is not clear. The purpose of the present study was to further examine the impact of HIGT on acute care LOS and change in Activity Measurement for post-acute care (AM-PAC 6-Clicks). **Methods**: This was a retrospective medical record review of a cohort of patients in the acute care setting who received HIGT following implementation of a quality improvement project. Historical conventional care patients were identified and matched to the HIGT group using propensity score matching. Linear mixed models were used to examine the effect of group on acute care LOS and change in AM-PAC 6-Clicks. **Results**: 36 HIGT patients with one matched control and 22 with two matched controls were analyzed. After controlling for unbalanced covariates using a linear mixed model, acute care LOS was 1.2 days shorter in the HIGT group compared to the conventional care control group (*p* = 0.031, 95% CI 0.11, 2.29). There was no difference in change in AM-PAC 6-Clicks (*p* = 0.575, 95% CI −1.36, 0.76). **Conclusions**: Receiving HIGT in the acute care setting was associated with a 1.2-day-shorter LOS compared to conventional care in this study cohort; however, exposure to HIGT does not appear to improve the functional status during acute hospitalization post-stroke in this cohort. The mechanism underlying the association with the shorter LOS remains unclear and may reflect increased confidence in the patient’s readiness for discharge, unmeasured differences between cohorts, or changes in a variety of factors that influence discharge disposition given the cohorts were from two different time periods.

## 1. Introduction

Stroke is a leading cause of disability in the United States with up to 50% of survivors having permanent disabilities and experiencing significant impairments in speech, cognition, motor function, sensory function, as well activity limitations in functional mobility and activities of daily living [1,2]. Approximately 80% of stroke survivors will experience activity limitations in walking, with recovery of walking function being critical to improving quality of life [1,3,4]. While many progress to independence, approximately 25% still require assistance with walking up to one year later [4]. For those who achieve independence, limitations in gait speed and endurance often persist. A significant contributor to chronic walking limitations is premature discontinuation of rehabilitation [4]. Additionally, conventional approaches to stroke rehabilitation may be insufficiently dosed to maximize recovery of walking function [5]. Conventional therapy only produces an average of about 250 steps per session, with the target heart rate being achieved less than 5% of the time [6].

In contrast, high-intensity gait training (HIGT) maximizes the time spent walking with a goal of achieving 75–85% of age-predicted heart rate maximum. If on beta-blockers, the rating of perceived exertion scale should be utilized with a target of 15–18/20 [7]. HIGT can be effective in patients with chronic stroke when initiated 6 months post-stroke [8]. However, intensive rehabilitation should start much earlier when the potential for neuroplasticity is greatest [9]. When used in the inpatient rehabilitation setting, HIGT produces results in a significantly higher daily step count [6,7]. However, little is known about initiating HIGT in the acute care setting.

Given the profound reductions in physical activity post-stroke in the acute care setting [10], early rehabilitation (48 h to two weeks post-stroke) is associated with improved clinical outcomes [11]. The evidence of the safety of intensive very early intervention (within 24 h post-stroke) is conflicting [12,13]. One of the most important factors associated with the discharge home is shorter time to initiation of mobilization and conventional physical therapy (PT) post-stroke [14]. Traditionally, PT in the acute care setting focuses on functional mobility and activities of daily living [4]. More recently, Thrush et al. observed that a greater number of PT visits and greater overall PT session duration were independently predictive of improvement in functional status by acute care discharge [15].

A more recent line of investigation into early intensive stroke rehabilitation in the acute care setting is HIGT. Two prior studies have demonstrated its feasibility and potential impact on function prior to acute care discharge [16,17]. Mulvaney et al. [17] noted improvements in gait and balance exceeding the minimum clinically important difference in a case series of five patients, with the target intensity being reached in half of the sessions. In a retrospective medical record review of 159 patients, Shoemaker et al. [16] similarly observed that target intensity was met approximately half the time and that earlier timing and greater dosing (HIGT sessions × session duration) were independent predictors of greater improvement in Activity Measurement for post-acute care (AM-PAC 6-Clicks) scores but did not have an impact of discharge disposition. However, there are two important caveats to their preliminary analysis: It only included patients who received HIGT (no control or comparator group), and the change in AM-PAC 6-Clicks was measured from the start of initiation of HIGT and not from admission to discharge [16].

Therefore, the purpose of the present study was to further examine the impact of HIGT on acute care LOS and change in AM-PAC 6-Clicks using a propensity score-matched historical, conventional care comparator group. The null hypotheses were that there would be no difference in acute care LOS or improvement in AM-PAC 6-Clicks. A secondary purpose was to describe the use of HIGT in the acute care setting and examine safety data in a third cohort beyond those studied by Shoemaker et al. and Mulvaney et al. [16,17].

## 2. Materials and Methods

### 2.1. Study Design and Participants

The present study utilized a retrospective medical record review of patients admitted for acute stroke to a Joint Commission-certified comprehensive stroke center. Patients in the HIGT intervention group were patients admitted between 1 January 2025 and 31 December 2025. Patients in the historical, conventional care comparator group were patients admitted between 1 August 2022 and 31 July 2023. Patients who received HIGT between 1 August 2023 and 31 December 2024 are described elsewhere [16]. The study was approved by the institutional review board (CHW-IRB-2024-0580).

Patients receiving HIGT did so as part of a recently implemented standard of care designed to maximize the time and intensity of gait and stepping practice. HIGT was provided to patients who were sufficiently stable based upon clinical judgment and required physical assistance of one to two people to stand and take steps. Although the facility does not use rigid criteria to determine stability, physical therapists consider values and trends in vital signs, cardiac rhythm, and laboratory values, pending imaging or procedures suggestive of suspected new or worsening pathology, new or worsening neurologic findings, and overall clinical trajectory as assessed by the interprofessional team. Patients who did not require any assistance to stand and take steps (i.e., who were independent with ambulation) did not receive HIGT.

Potentially eligible records for inclusion were identified based on an internally maintained list of patients who received HIGT. The target session duration was 25 min with a target intensity of 75–85% of age-predicted maximum heart or a rating of perceived exertion of 15–18/20 for those on chronotropic medications. Patients in the HIGT group were included if they received at least one HIGT session.

Patients in the historical, conventional care comparator group were included if they received at least one PT session. Potential records for inclusion into the conventional care group were identified using an internal, custom-written, electronic health record search algorithm based on ICD-10 code and admission date. The search was then further refined to identify patients with the data required for propensity score matching. The goal was to efficiently identify, through electronic search, as many possible conventional care group patients as a manual search and data extraction would not have been feasible. Patients in both groups were required to have been admitted with acute ischemic or hemorrhagic stroke and were excluded if they had any new significant complications post-admission that would confound the length of stay. Each reason for exclusion was discussed by the research team to ensure the criteria were applied equally between groups.

Data collected included demographic and clinical characteristics, functional status using the AM-PAC 6-Clicks, length of stay (LOS), number and timing of PT visits and session duration, adverse events, and characteristics of HIGT sessions. AM-PAC 6-Clicks scores from the first and last PT visit were used. Adverse events were conservatively defined as any documented unexpected event or change in status during, immediately following, or within 24 h of a PT visit, without determination of the cause or relationship to the PT visit or interventions used.

### 2.2. Statistical Analysis

Descriptive statistics were calculated for demographic and clinical characteristics for the HIGT group and the propensity score-matched historical conventional care comparator group. Potential historical conventional care comparator records were identified using an automated electronic health record search as noted above. This search precluded the ability to use any detailed clinical characteristics or clinical context as these data were not able to be electronically searched and a manual data extraction prior to matching on a large volume of patients was not feasible. Therefore, the only relevant demographic and clinical characteristic variables available for propensity score matching were stroke type, sex, age, body mass index (BMI), number of PT visits, and initial AM-PAC 6-Clicks.

Propensity score matching consisted of logistic regression with exact matching for stroke type (ischemic or hemorrhagic) and number of PT visits, as well as nearest neighbor matching without replacement and a propensity score match tolerance of 0.2 for age and initial AM-PAC 6-Clicks. Because initial matching was based on this limited set of variables and some matched comparators were anticipated to be excluded following a detailed manual chart review and identification of one or more exclusion criteria, 2:1 matching was used to allow for identification of a second comparator for matching to minimize the number of HIGT patients without a match. Matching balance statistics were re-calculated for the final, matched sample and are summarized in Table 1.

Although weighted bivariate, between-group comparisons for the HIGT and propensity score-matched conventional care groups were used to compare baseline demographic and clinical characteristics for descriptive purposes, the variable matching ratios (due to missing data/dropped cases) required the use of linear mixed models to examine the effect of group on acute care LOS and change in AM-PAC 6-Clicks. The use of linear mixed modeling also allowed for a “doubly robust” treatment effect estimation by adding clinical characteristics that were no longer balanced (i.e., standardized mean difference >0.10) as covariates.

Multivariate linear regression was used to examine the effect of PT intervention dose (i.e., total number of PT visits) on change in AM-PAC 6-Clicks while controlling for acute care LOS. Acute care LOS was entered in step 1, total PT visits in step 2, and group in step 3. SPSS statistical software version 26 (RRID:SCR_002865) was used for data analysis.

## 3. Results

**Figure 1:**
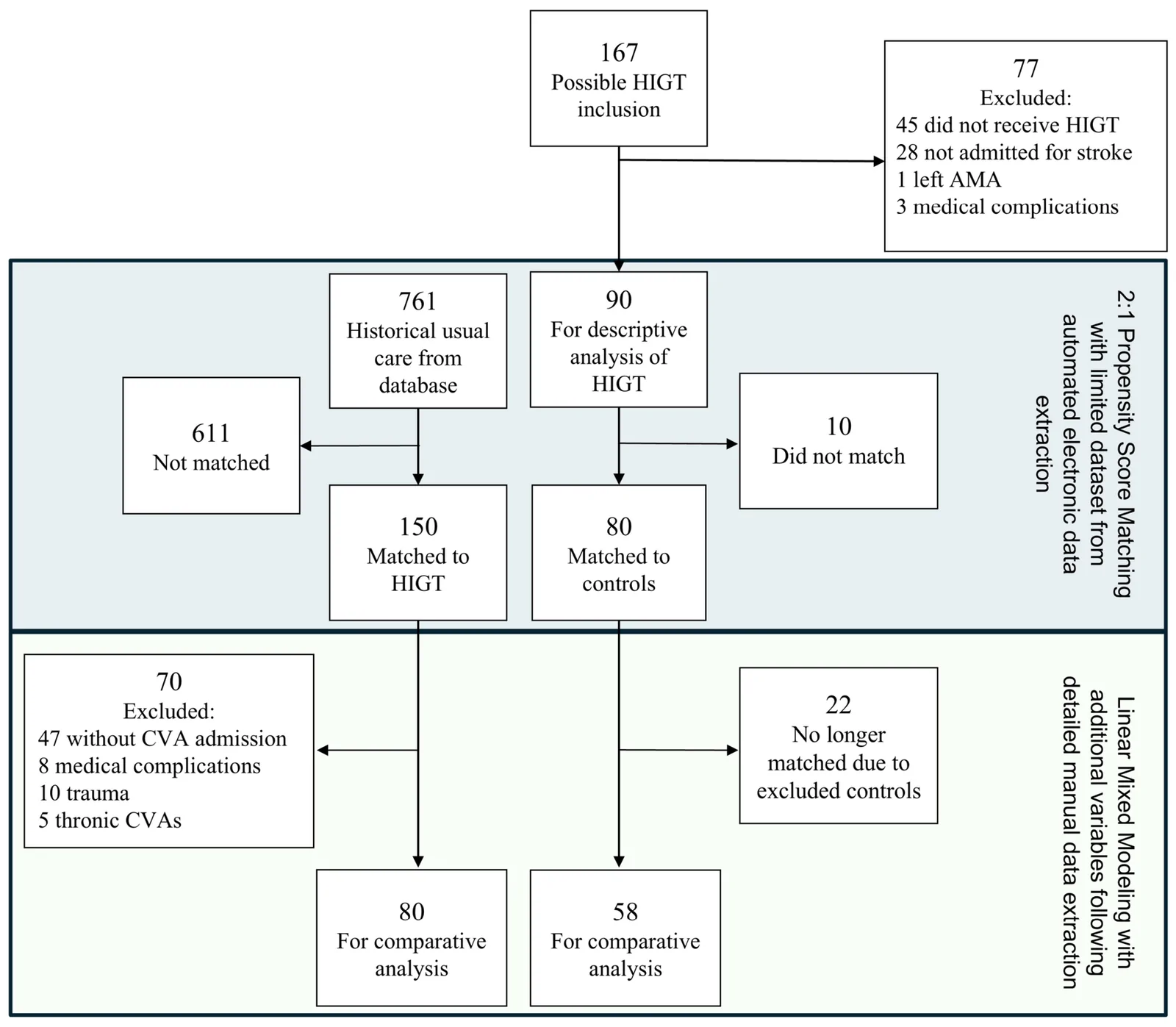
Flow diagram for the high-intensity gait training (HIGT) and historical conventional care groups. CVA = cerebrovascular accident.

Regarding the HIGT group, a total of 167 records were considered for inclusion with 90 included for descriptive analysis. A total of 80 matched pairs were initially identified (12 with one matched control and 68 with two matched controls). However, upon manually collecting detailed additional data for the conventional care group, 70 were found to meet exclusion criteria, resulting in 58 patients who received HIGT and 80 control patients who received conventional care. The 58 matched sets for analysis comprised 36 with one matched control and 22 with two matched controls (Figure 1).

Descriptive statistics of the sample are provided in Table 2 and Table 3 and are stratified by group and stroke type. The majority of patients had cortical/deep brain ischemic stroke. There were no significant differences in any baseline demographic and clinical characteristics between the HIGT and conventional care groups using weighted *t*-tests and chi square analyses.

### 3.1. Descriptive Analysis of HIGT

HIGT was initiated at an average of 4.7 days after hospital admission while acute care LOS was an average of 8.5 days indicating that HIGT treatment started just over halfway during hospital stay. Upon initiation of HIGT, mean AM-PAC 6-Clicks was 14.8. The HIGT group had a mean of 4.9 total PT visits, with a mean of 2.3 of those visits using HIGT. Target intensity was met 36.7% of the time.

Table 4 describes the HIGT intervention. Among patients who received HIGT, 21.1% initially required assistance from more than one person and 71.6% of patients used a wheeled walker during HIGT. Limb advancement (76.6%) and propulsion (78.8%) were the gait determinants most frequently emphasized, with a wide range of specific intervention strategies utilized during all HIGT visits.

Across the 207 HIGT sessions studied, four of the 90 patients (4.4% patients, 1.9% of HIGT sessions) had an adverse event within 24 h of a HIGT session (Table 5). Regarding the safety of HIGT, only one adverse event was temporally associated with and therefore plausibly attributable to HIGT as it occurred shortly after a PT session (hypotension requiring medication change and monitoring) but did not result in an overall change in clinical status. The other three adverse events were only temporally associated with but not plausibly attributable to HIGT. Additionally, there were no falls during any HIGT session.

### 3.2. Comparison of HIGT Cohort vs. Historical Conventional Care Cohort

Matching balance for the initially matched pairs was excellent with all standardized mean differences less than 0.05 except initial AM-PAC 6-Clicks (0.058). Following attrition of cases and matched sets due to the exclusion of initially matched controls, balance statistics for the final, matched sample used for all comparative analyses were calculated for Charleson Comorbidity Index, BMI, and peak NIHSS score, and were re-calculated for the age, total PT visits, and initial AM-PAC 6-Clicks score. Although there were no significant differences in any baseline demographic and clinical characteristics between the HIGT and conventional care groups based on weighted bivariate analyses, standardized mean differences greater than 0.10 were observed for the Charleson Comorbidity Index (0.24), total PT visits (0.16), initial AM-PAC 6-Clicks score (0.18), and peak NIHSS score (0.29). Therefore, and as previously noted, the linear mixed models included these four variables as covariates (Table 1).

When controlling for Charleson Comorbidity Index, total PT visits, initial AM-PAC 6-Clicks score, and peak NIHSS score using linear mixed modeling, the acute care LOS was 1.2 days shorter in the HIGT group compared to the historical conventional care control group (*p* = 0.031, 95% CI 0.11, 2.29) (Table 6). However, there was no difference in the change in AM-PAC 6-Clicks (*p* = 0.575, 95% CI −1.36, 0.76) (Table 7).

When qualitatively comparing adverse events, the historical conventional care group was comparable to the HIGT group with five adverse events (6.3% of patients, 2.0% of PT visits) and only one complication occurred during or immediately following PT (vs. 4.4% patients, 1.9% of sessions, and one during or immediately following a HIGT session). The conventional care patient with an adverse event during or immediately following the PT session developed sluggish pupils and enlargement of the third ventricle, which was managed with close monitoring and temporary withholding of anticoagulant and antiplatelet medications in anticipation of possible surgical intervention; however, no surgical treatment was ultimately required.

Regarding the effect of dose/total number of visits and group on change in AM-PAC 6-Clicks while controlling for acute care LOS using multivariate linear regression, only the total PT visits, but not group, was a statistically significant predictor of change in function (overall model adjusted R^2^ = 0.11, total PT visits β = 0.42, *p* < 0.001, group β = −0.02, *p* = 0.852).

## 4. Discussion

The present study sought to compare the differences in acute care LOS and change in AM-PAC 6-Clicks between a HIGT cohort and a historical conventional PT intervention, as well as describe the use of HIGT in the acute care setting and examine safety data in an additional cohort beyond those previously studied [16,17]. The results of the present study indicate that although there was an association between receiving HIGT and a shorter length of stay, there does not appear to be an effect of HIGT on improvement in functional status. However, there are some important limitations related to HIGT dosing and outcome measurement that must be considered and are discussed below. Additionally, the low number of adverse events in those receiving HIGT provides additional evidence that it is safe and can be used in the acute care setting in patients with stroke with an AM-PAC 6-Clicks score between six and 22, with most having scores between eight and 18.

Regarding the association between receiving HIGT and a shorter length of stay, it is not clear why this treatment effect was observed given there was no difference in the change in functional status and when there was a trend of more patients in the HIGT group who were discharged to IPR. This observation could be due to several possibilities. First, HIGT may increase the interprofessional team’s confidence about the patient’s readiness for discharge from a functional and/or medical stability perspective resulting in earlier discharge to an IPR setting.

A second possibility is that the analysis of acute care LOS could be confounded by a variety of other factors influencing discharge disposition. For example, although not statistically significant (X^2^ = 5.25, *p* = 0.263), there tended to be more patients in the HIGT group who were initially recommended for inpatient rehabilitation (IPR) discharge and who were then discharged to IPR. As noted above, their performance with HIGT may have then facilitated earlier discharge to that setting which was already projected for that patient.

A third most likely and problematic possible explanation is that there are numerous factors that affect discharge disposition, including clinician preferences, health care system priorities and initiatives, patient and family preferences, and payer policies. Given that the historical conventional care group was from a time frame nearly two years prior to the HIGT group, any of these confounding factors could have changed over time. However, this observed shorter LOS for the HIGT group is an important preliminary, hypothesis-generating result which, regardless of the hypothesized mechanism, clearly indicates the need for further study given the relatively wide 95% confidence interval of 0.11 to 2.29 days. Contemporaneous controlled studies are the necessary next step.

Regarding the lack of effect of HIGT on improvement in AM-PAC 6-Clicks, this was an unexpected finding. It could be explained by the relatively late start of HIGT (at a mean of 4.7 days into an 8.5-day LOS), a mean of only 2.3 HIGT sessions, and that the target intensity was only met 36.7% of the time. Although Shoemaker et al. [16] found a dose–response relationship between the HIGT dose (visits × visit duration) and improvement in AM-PAC 6-Clicks when controlling for acute care LOS, there was no comparator group to contextualize the importance of that observation. The inclusion of a comparator group and controlling for the number of PT visits in the present study may indicate that the previously observed dose–response relationship is a more general dose–response relationship of PT sessions regardless of intervention provided. Indeed, the total PT visits, but not group, was a predictor of change in AM-PAC 6-Clicks. This is consistent with previous studies demonstrating greater number of PT visits and session duration being predictive of improvement in the functional status by acute care discharge [15,18] and of discharge home [19], but not with Edelstein et al., where only timing of PT evaluation, but not therapy intensity, was associated with improvement in function at discharge [18]. This could be due to the low threshold for inclusion into the HIGT group (i.e., at least one HIGT session) and therefore an underdosing of HIGT. It is also possible that the short time frame of an acute care LOS may be too short to allow for clear evaluation of the effect of HIGT on improvement in functional status in the acute care study. In short, it may be more accurate to represent this HIGT group as a HIGT-exposed group. Additionally, the AM-PAC 6-Clicks is likely insufficiently sensitive to the changes in locomotor function that might occur during the relatively short time period of acute care hospitalization. However, due to the retrospective design and use of a historical comparator group, no additional functional performance data were consistently available for analysis. The results of the present study should be considered hypothesis-generating, as further study is clearly warranted to determine the minimum exposure and intensity of HIGT required to impact gait-specific outcome measures.

With regard to the delivery and safety of HIGT in the acute care setting, the findings are consistent with previous studies [16,17]. Based on retrospective analysis, there does not appear to be an increased safety risk in the present patient cohort who were sufficiently medically stable for PT intervention, and who had low-to-moderate levels of physical functioning (i.e., AM-PAC 6-Clicks of 8–18) who need assistance of one or two people, with or without a basic assistive device, to stand and take steps. However, achieving the target intensity in the acute care setting is difficult and is similar to that observed in other settings [6]. Future research is needed to prospectively and systematically examine safety, retention and drop-out rates, completion of intended interventions, protocol adherence, patient perception and satisfaction, and clinical staff burden and costs.

Regarding potential threats to internal validity, several limitations should be considered. First and most importantly, HIGT was generally under-dosed with respect to timing of initiation, number of sessions, and meeting intensity targets. This, combined with the use of the AM-PAC 6-Clicks as an outcome measure that may not be sensitive to subtle changes in locomotor function, limits its ability to assess the impact of HIGT on function during the short time frame of an acute care hospitalization. Second, number of PT visits was used for matching and was again used in linear mixed modeling which could confound the analysis of the length of stay given their inherent association. However, controlling for the PT dose was critical as matching patients with vastly different doses of PT would preclude any conclusions about the type of PT intervention (HIGT vs. conventional). Third, there was potential for selection bias due to the methods used to identify potential records for inclusion. For patients in the historical conventional care group, use of stroke-related ICD-10 code stems for admission diagnosis resulted in numerous records that ultimately excluded and affected the overall size of the matched sample. Additionally, records for the HIGT group were identified using a quality improvement tracking list maintained by treating physical therapists which relied upon therapists consistently using the tracking sheet and therefore all patients who received HIGT may not have been identified. Further, confounding by indication is also a risk due to the non-systematic selection through reliance upon clinician judgment. A fourth threat to internal validity is the loss in cases in the conventional care control group following initial matching based solely on automatically collected data. The exclusion of records based on the detailed manual record review could have introduced additional selection bias. A fifth threat to internal validity was the data available for controlling for stroke severity. Uniform availability of NIHSS scores at various time points (admission, 24 h post-stroke, at time of PT evaluation, etc.) was poor and would have resulted in a considerable loss in the sample size. Therefore, peak NIHSS was used as it was the best estimate of severity available across all patients.

Regarding threats to external validity, generalizability may be limited due to the relatively small sample or primarily those with ischemic stroke from a single institution which may not represent the broader stroke population or practice patterns at other institutions.

## 5. Conclusions

A description of the delivery and safety of HIGT in the acute care setting was again reported in the present study. Compared to conventional PT intervention in a historical cohort, HIGT within the acute care setting for patients with acute stroke was associated with a 1.2-day-shorter LOS in this HIGT cohort. Given that HIGT was not associated with greater improvement in functional status, the mechanism by which the shorter LOS was observed and the longer-term impact of HIGT remains unknown and requires further study using contemporaneous controlled studies.

## Figures and Tables

**Table 1:** Between-group standardized mean differences for variables included in statistical modeling.

	Standardized Mean Differences		
	Before Propensity Score Matching	After Propensity Score Matching	After Exclusions Following Detailed Manual Data Extraction
**Propensity Score**	0.206	0.036	-
**Stroke Type**	0.284	−0.028	-
**Age**	−0.188	−0.012	-
**Total Physical Therapy Visits**	0.247	0.000	0.156 ^#^
**Baseline AM-PAC 6-Clicks**	0.127	0.058	0.178 ^#^
Stroke Type *	-	-	0.054
Age *	-	-	−0.067
Charleson Commodity Index *	-	-	0.235 ^#^
Peak NIHSS *	-	-	0.285 ^#^
Body Mass Index *	-	-	0.091

* = variable not available for automated electronic data extraction for control group; ^#^ = variable included in linear mixed modeling; AM-PAC 6-Clicks = Activity Measurement for post-acute care; NIHSS = national institutes of health stroke scale.

**Table 2:** Clinical characteristics of matched patients.

	Ischemic		Hemorrhagic	
	HIGT (N = 52)	Control (N = 73)	HIGT (N = 6)	Control (N = 7)
**Clinical Characteristics**				
Age (years)	69.6 (14.3)	69.3 (14.4)	66.0 (16.0)	57.6 (21.5)
Sex (male)	29 (55.8%)	35 (47.9%)	4 (66.7%)	4 (57.1%)
Initial AM-PAC 6-Clicks (score)	14.0 (4.2)	13.8 (4.6)	14.3 (3.3)	11.6 (4.8)
Final AM-PAC 6-Clicks (score)	16.9 (3.3)	16.1 (4.6)	16.3 (1.9)	15.1 (6.2)
Stroke Location (cortical/deep brain)	47 (92.2%)	47 (64.4%)	6 (100%)	4 (57.1%)
Aphasia (yes)	15 (28.9%)	19 (26.0%)	0 (0.0%)	2 (28.6%)
Body Mass Index (kg/m^2^)	28.4 (5.8)	29.2 (6.7)	30.4 (7.2)	28.8 (8.4)
Charlson Comorbidity Index (score)	5.0 (2.4)	5.7 (2.4)	5.8 (3.4)	3.7 (2.2)
**Stroke Severity**				
Peak NIHSS	8.7 (6.3)	10.2 (8.0)	4.2 (1.8)	6.5 (3.5)
**Description of Intervention**				
Number of Physical Therapy Visits	3.6 (2.0)	3.2 (1.5)	3.0 (1.1)	2.9 (1.1)
Number of HIGT Visits	1.7 (1.0)	0.0 (0.0)	1.5 (0.8)	0.0 (0.0)
**Clinical Outcomes**				
Length of Stay (days)	6.4 (4.7)	7.0 (4.8)	5.0 (2)	6.7 (2.6)
Actual Discharge Location				
Home	8 (15.4%)	22 (30.1%)	0 (0.0%)	3 (42.9%)
Inpatient Rehabilitation	41 (78.8%)	33 (45.2%)	4 (66.7%)	3 (42.9%)
Subacute	3 (5.8%)	18 (24.7%)	2 (33.3%)	1 (14.3%)
Improved Discharge Disposition	4 (7.7%)	13 (17.8%)	0 (0%)	2 (28.6%)

Values are mean (standard deviation) or number (percent); kg/m^2^, kilograms per meters squared; LOS, length of stay; HIGT, high-intensity gait training; NIHSS, national institutes of health stroke scale; AM-PAC 6-Clicks, activity measure for post-acute care.

**Table 3:** Clinical characteristics of all patients receiving high-intensity gait training (before matching).

	All (N = 90)	Ischemic (N = 72)	Hemorrhagic (N = 18)
Clinical Characteristics			
Age (years)	67.3 (14.8)	67.3 (15.2)	67.4 (13.9)
Sex (male)	49 (54.4%)	41.0 (56.9%)	8 (44.4%)
Initial AM-PAC 6-Clicks (score)	13.1 (4.6) 7 (7.8%) score = 6 0 (0.0%) score = 24	13.5 (4.6) 4 (5.6%) score = 6 0 (0.0%) score = 24	11.4 (4.3) 3 (16.7%) score = 6 0 (0.0%) score = 24
Final AM-PAC 6-Clicks (score)	16.0 (3.9) 3 (3.3%) score = 6 2 (2.2%) score = 24	16.3 (3.7) 2 (2.8%) score = 6 2 (2.8%) score = 24	14.9 (4.2) 1 (5.6%) score = 6 0 (0.0%) score = 24
Aphasia (yes)	30 (33.3%)	24.0 (33.4%)	6.0 (35.2%)
Body Mass Index (kg/m^2^)	28.3 (5.9)	27.8 (5.8)	30.2 (6.1)
Charlson Comorbidity Index (score)	5.0 (2.4)	5.0 (2.4)	5.2 (2.7)
**Stroke Severity**			
Peak NIHSS	9.4 (7.2)	9.3 (7.2)	9.8 (7.7)
**Description of Intervention**			
Number of Physical Therapy Visits	4.9 (4.1)	4.6 (3.6)	6.1 (5.4)
Number of HIGT Visits	2.3 (2.4)	2.3 (2.4)	2.5 (2.2)
Days to Start of HIGT (days)	4.7 (4.2)	4.1 (2.6)	7.1 (7.4)
AM-PAC 6-Clicks at Start of HIGT	14.8 (4.0)	15.1 (3.9)	13.3 (4.3)
HIGT Dose (visits × time)	56.1 (76.9)	56.3 (81.3)	55.4 (56.0)
Intensity Target (met)	33 (36.7%)	26 (36.1%)	7 (38.9%)
Duration of HIGT (minutes)	22.5 (8.6)	22.9 (9.1)	20.6 (6.6)
**Clinical Outcomes**			
Δ AM-PAC 6-Clicks (from first HIGT session)	2.9 (3.4)	2.8 (3.5)	3.6 (3.4)
Length of Stay (days)	8.5 (8.4)	7.6 (6.2)	12.4 (13.6)
Actual Discharge Location			
Home	12 (13.3%)	10 (13.9%)	2 (11.1%)
Inpatient Rehabilitation	65 (72.2%)	53 (73.6%)	12 (66.7%)
Subacute	13 (14.4%)	9 (12.5%)	4 (22.2%)
Δ Discharge Disposition (improved)	7 (7.8%)	5 (7.0%)	2 (11.1%)

Values are mean (standard deviation) or number (percent); kg/m^2^, kilograms per meters squared; LOS, length of stay; HIGT, high-intensity gait training; NIHSS, national institutes of health stroke scale; AM-PAC 6-Clicks, activity measure for post-acute care.

**Table 4:** Description of high-intensity gait training intervention implementation.

Intervention Element	Description	
**Assistance**	19 of 90 (21.1%) required assist of 2	
**Assistive Device**	23 of 90 (25.55%) required no device Of those, 2 (2.2%) used hand hold assist. 67 of 90 (74.4%) initially used an assistive device. Of those: 11 (16.4%) cane (single point or quad); 48 (71.6%) wheeled walker; 6 (8.9%) specialized gait trainer equipment.	
**Determinants of Gait**	Determinants of gait that were targeted were (not mutually exclusive): 71 (78.8%) propulsion; 69 (76.6%) limb advancement; 66 (73.3%) lateral stability; 64 (71.1%) stance control.	
**Examples of Specific Interventions**	dual task head turns stop and go direction changes obstacle navigation quick stops environmental scanning high knees tandem walk visual step targets step length gait normalization gait without assistive device	speed changes stairs retro ambulation perturbations lateral stepping step ups 180 degree turns pick up items on floor eyes closed weight shifts resisted walking whole task progress gait distance

Values are number (percent).

**Table 5:** Adverse Events.

Adverse Event	Conventional Care	High-Intensity Gait Training
**Headache**	**One during session:** Headache that developed during physical therapy session managed pain medication; no change in clinical status **One within 24 h:** Migraine that developed five hours following physical therapy session managed with migraine medications; no change in clinical status	
**Respiratory Failure**	**One within 24 h:** Intubation 20 h following physical therapy session due to respiratory failure not consistent with PT session	
**Change in Vital Signs**		**Two within 24 h:** High blood pressure 24 h after physical therapy session, managed with medication, vitals and imaging unchanged Became hypotensive and lethargic one hour following physical therapy session, managed with medication and monitoring, vitals remained stable
**Other**	**One within 24 h:** Sluggish pupils and enlarged 3rd ventricle managed through close monitoring and hold on anticoagulants/antiplatelets in case of surgical intervention (but surgery not needed)	**Two within 24 h:** Change in cognition following physical therapy visit although returned to baseline cognition same day; vitals remained stable None except witnessed assisted fall five hours after physical therapy session; vitals remained stable, no change in imaging

**Table 6:** Linear mixed model parameters for length of stay.

Parameter	Estimate	Standard Error	Significance	95% Confidence Interval
Intercept	−3.35	1.90	0.063	−6.89, 0.18
Group (Control) *	1.20	0.55	0.031	0.11, 2.29
Initial AM-PAC 6-Clicks	0.08	0.08	0.314	−0.08, 0.23
Charleson Comorbidity Index	0.08	0.11	0.451	−0.14, 0.30
Total PT Visits	2.09	0.19	<0.001	1.72, 2.45
Peak NIHSS	0.09	0.04	0.047	0.00, 0.18

* Estimated Marginal Means (95% Confidence Interval) for length of stay for controls and HIGT groups, respectively: 7.1 (6.4, 7.8) vs. 5.9 (5.1, 6.7); AM-PAC 6-Clicks, activity measure for post-acute care, NIHSS, national institutes of health stroke scale.

**Table 7:** Linear mixed model parameters for change in AM-PAC 6-Clicks.

Parameter	Estimate	Standard Error	Significance	95% Confidence Interval
Intercept	6.64	1.76	<0.001	3.16, 10.12
Group (Control) *	−0.30	0.54	0.575	−1.36, 0.76
Initial AM-PAC 6-Clicks	−0.35	0.08	<0.001	−0.50, −0.20
Charleson Comorbidity Index	−0.08	0.11	0.505	−0.29, 0.14
Total PT Visits	0.45	0.18	0.015	0.09, 0.81
Peak NIHSS	−0.03	0.04	0.480	−0.12, 0.06

* Estimated Marginal Means (95% Confidence Interval) for change in AM-PAC 6-Clicks for controls and HIGT groups, respectively: 2.3 (1.6, 3.0) vs. 2.6 (1.8, 3.4); AM-PAC 6-Clicks, activity measure for post-acute care, NIHSS, national institutes of health stroke scale.

## Data Availability

The data presented in this study are available on request from the corresponding author because public data sharing was not included in the IRB-approved protocol.

## Abbreviations

The following abbreviations are used in this manuscript:

HIGT	High-intensity gait training
PT	Physical therapy
AM-PAC 6-Clicks	Activity Measurement for post-acute care
LOS	Length of stay
BMI	Body mass index
NIHSS	National Institutes of health stroke scale
IPR	Inpatient rehabilitation
